# Accurate and Rapid Identification of the *Burkholderia pseudomallei* Near-Neighbour, *Burkholderia ubonensis*, Using Real-Time PCR

**DOI:** 10.1371/journal.pone.0071647

**Published:** 2013-08-13

**Authors:** Erin P. Price, Derek S. Sarovich, Jessica R. Webb, Jennifer L. Ginther, Mark Mayo, James M. Cook, Meagan L. Seymour, Mirjam Kaestli, Vanessa Theobald, Carina M. Hall, Joseph D. Busch, Jeffrey T. Foster, Paul Keim, David M. Wagner, Apichai Tuanyok, Talima Pearson, Bart J. Currie

**Affiliations:** 1 Global and Tropical Health Division, Menzies School of Health Research, Darwin, Northern Territory, Australia; 2 Center for Microbial Genetics and Genomics, Northern Arizona University, Flagstaff, Arizona, United States of America; Tulane University School of Medicine, United States of America

## Abstract

*Burkholderia ubonensis* is an environmental bacterium belonging to the *Burkholderia cepacia* complex (Bcc), a group of genetically related organisms that are associated with opportunistic but generally nonfatal infections in healthy individuals. In contrast, the near-neighbour species *Burkholderia pseudomallei* causes melioidosis, a disease that can be fatal in up to 95% of cases if left untreated. *B. ubonensis* is frequently misidentified as *B. pseudomallei* from soil samples using selective culturing on Ashdown’s medium, reflecting both the shared environmental niche and morphological similarities of these species. Additionally, *B. ubonensis* shows potential as an important biocontrol agent in *B. pseudomallei-*endemic regions as certain strains possess antagonistic properties towards *B. pseudomallei*. Current methods for characterising *B. ubonensis* are laborious, time-consuming and costly, and as such this bacterium remains poorly studied. The aim of our study was to develop a rapid and inexpensive real-time PCR-based assay specific for *B. ubonensis.* We demonstrate that a novel *B. ubonensis-*specific assay, Bu550, accurately differentiates *B. ubonensis* from *B. pseudomallei* and other species that grow on selective Ashdown’s agar. We anticipate that Bu550 will catalyse research on *B. ubonensis* by enabling rapid identification of this organism from Ashdown’s-positive colonies that are not *B. pseudomallei.*

## Introduction

The Gram-negative *Burkholderia* spp. comprise an ecologically diverse group containing over 70 species (http://www.bacterio.cict.fr/b/burkholderia.html), some of which are pathogenic to humans, animals or plants [1], [2]. *Burkholderia pseudomallei* is the best-known member of the genus due to its ability to cause the potentially fatal disease melioidosis [3] and its biothreat potential [4]. *B. pseudomallei* was recently added as a Tier 1 Select Agent in the United States, a category that includes those organisms of greatest threat to human and animal health. *B. pseudomallei* is commonly recovered in the environment in northern Australia (particularly the “Top End“ of the Northern Territory) and north-eastern Thailand, but has also been described from a much wider endemic region including most other countries in Southeast Asia, the Indian subcontinent, Taiwan, southern China and Hong Kong [5]. The presence of *B. pseudomallei* in Africa and the Americas has also been described but the extent of its distribution remains unclear [6]. Several other soil-dwelling *Burkholderia* spp. reside in ecological niches where *B. pseudomallei* is present, and some of these species can also cause opportunistic, albeit less serious, infections in humans. Many of these species fall into the *Burkholderia cepacia* complex (Bcc), which contains at least 17 *Burkholderia* species, including *Burkholderia ubonensis* [7].

Misidentification of *Burkholderia* spp. has implications for environmental studies, clinical diagnosis and biosecurity responses [8], especially for *B. pseudomallei*, where false-negative and false-positive results may have serious consequences. Species misidentification can have an economic impact, as demonstrated by false-positive calls of near-neighbour species under the BioWatch program, which was introduced in the United States in 2003 to monitor aerosol samples for the presence of Select Agent organisms in the environment [9]. Detecting *B. pseudomallei* from clinical samples is also a nontrivial endeavour. Most hospital laboratories use standard culture media (e.g. MacConkey, horse blood and chocolate agars) for culturing of clinical specimens. Morphological identification of *B. pseudomallei* in non-endemic areas is therefore difficult due to unfamiliarity, a lack of selective media available for identification [10], and the frequent misidentification of *B. pseudomallei* using automated systems such as VITEK 2 [11]. In endemic regions, *B. pseudomallei* is typically enriched from environmental specimens using broth selection [12] followed by plating on Ashdown’s agar (ASA) [13]. However, no selective method is *B. pseudomallei*-specific. Indeed, many *Burkholderia* spp. residing in the same niches as *B. pseudomallei*, including *B. ubonensis*, are morphologically similar on ASA [10], [14], [15].

Since the “*Burkholderia uboniae*” species was first proposed in 2000 [16], little research has been conducted on *B. ubonensis*, despite being a potentially important biocontrol agent for *B. pseudomallei* [15]. Dideoxy sequencing-based genotyping approaches such as multilocus sequence typing (MLST), *recA* and 16S sequencing have been developed for *Burkholderia* spp. characterisation [8], [17], [18]. However, there are currently no cost-effective, rapid, and simple methods for detecting and differentiating *B. ubonensis* from other *Burkholderia* spp. including *B. pseudomallei*. For example, the type III secretion system 1 (TTS1) assay [19] only detects *B. pseudomallei*, and thus cannot further identify other species that grow on ASA. Therefore, the major aim of our study was to differentiate *B. ubonensis* from *B. pseudomallei*, with a secondary aim of differentiating *B. ubonensis* from other members of the Bcc and non-Burkholderiaecae organisms that also grow on ASA.

## Materials and Methods

### Ethics Statement

The Australian isolates used in our study were obtained from either private land or from Aboriginal communities. Prior to private land soil and water sampling, we obtained signed or verbal permission from land owners. Sampling permits were obtained from Northern Land Council (Northern Territory, Australia) prior to sample collection from Aboriginal communities. As per permit conditions, we obtained further permission from the community representatives prior to sampling. No specific permits were required for collection of the Thai isolates as they were obtained from unregulated public lands. Our field collection did not involve endangered or protected species.

### Bacterial Isolates

Our laboratories have ongoing collections of isolates from soil and water samples obtained from both the Northern Territory and Thailand, comprising isolates that grow on ASA [13] yet are not *B. pseudomallei* according to the TTS1 assay [19]. These isolates were subjected to 16S sequencing, MLST, *recA* sequencing or whole-genome sequencing (WGS) as part of this and other studies to confirm genus and, where possible, for species assignment. All isolates were subcultured for purity on chocolate agar or ASA (Oxoid, Thebarton, SA, Australia) prior to DNA extraction. The Qiagen DNeasy kit (Qiagen, Doncaster, VIC, Australia) was used for DNA extraction as previously described [20]. DNA was diluted 1∶100 in molecular-grade H_2_O prior to PCR.

### Bioinformatic Analysis to Identify *B. ubonensis-*specific Loci

Nineteen *B. pseudomallei* near-neighbour isolates were subjected to WGS: *Burkholderia* spp. MSMB175, *Burkholderia* spp. MSMB49, *B. cenocepacia* MSMB101, *B. cenocepacia* MSMB139, *Burkholderia multivorans* MSMB104, *B. multivorans* MSMB105, *Burkholderia oklahomensis* C6786, *B. pseudomallei* MSHR684, *B. pseudomallei* MSHR1079, *Burkholderia thailandensis-*like strain MSMB121, *B. thailandensis* MSMB59, *B. ubonensis* MSMB56, *B. ubonensis* MSMB106, *B. ubonensis* MSMB108, *B. ubonensis* MSMB145, *B. ubonensis* MSMB157, *B. ubonensis* MSMB166, *B. ubonensis* MSMB169 and *B. ubonensis* MSMB170. The Illumina GAIIx platform (Illumina, San Diego, CA, USA) was used to generate WGS data. An assembly of *B. ubonensis* MSMB170 was performed on paired-end Illumina v1.9 reads with Velvet v1.2.07 [21], using a kmer of 55. This assembly resulted in 836 contigs with an n50 of 101,278 bp. MSMB170 was subsequently used as a reference genome for read mapping with the Burrows-Wheeler Aligner (BWA) v0.5.9 [22]. The coverageBed module of BEDTools v2.15.0 [23] was used for presence/absence analysis based on a 1 kb window size. Candidate *B. ubonensis-*specific loci were identified by locating regions with 100% read coverage in all eight *B. ubonensis* strains but with <50% coverage in other *Burkholderia* species. Eleven candidate loci ≥5 kb were identified. One locus, Bu550, was chosen for real-time PCR assay design following confirmation of *in silico* specificity for *B. ubonensis* using Microbial Nucleotide BLAST.

### *B. ubonensis-*specific Real-time PCR Assay Bu550

A fluorogenic probe-based real-time PCR assay (Bu550) was developed to target a candidate *B. ubonensis-*specific 7 kb locus. Four putative protein products are encoded within this locus; a major facilitator superfamily transporter (GenBank ID: WP_010089641), a hypothetical protein (WP_010089640), a carbamoyltransferase (WP_010089639) and a tannase (WP_010089638). Unlabelled primers Bu550-F (5′-ATGCCGTGATCGACAACGAT) and Bu550-R (5′-ACTCCAGAAACAGTTCAGGCGT) (Invitrogen, Mulgrave, VIC, Australia) were used to amplify a conserved 91-bp fragment within this locus. A Black Hole Quencher (BHQ) probe (5′-CAL Fluor Gold 540-CGGGTGATGTGGCGTGACATTTACAGA-BHQ1; Biosearch Technologies, Novato, CA, USA) was included to increase specificity. BLAST analysis was conducted on the primers and probes to ensure assay specificity and accuracy. Real-time PCR was performed in 384-well optical plates (Applied Biosystems, Foster City, CA, USA). Each 5 µL reaction contained 0.3 µM of each primer, 0.2 µM of probe, 1X TaqMan Environmental Master Mix (Applied Biosystems) and 1 µL genomic DNA, to a total reaction volume of 5 µL. We also tested 1X TaqMan Universal Master Mix (Applied Biosystems) to determine assay robustness across different mastermixes. The 306 isolates used in this study (Table 1) were tested in duplicate, and all runs contained appropriate positive control and no-template control reactions. Thermocycling was carried out under default conditions using an ABI PRISM 7900HT instrument (Applied Biosystems), using the TET channel for fluorescence detection.

**Table 1:** Bacterial strain panel used in this study.

Species	No. strains^a^
*Achromobacter* spp.	1
*Acidovorax caeni*	1
*Alcaligenes spp.*	1
*Burkholderia cenocepacia*	2 (1)
*Burkholderia cepacia*	2 (16)
*Burkholderia diffusa*	2 (1)
*Burkholderia multivorans*	3
*Burkholderia pseudomallei*	75 (11)
*Burkholderia pyrrocinia*	1
*Burkholderia thailandensis*	3
*Burkholderia thailandensis*-like^b^	2
*Burkholderia ubonensis*	125 (15)
*Burkholderia vietnamiensis*	1
*Burkholderia* spp. BCCU6	1
*Burkholderia* spp. M279	2
*Chromobacterium violaceum*	1
*Chryseobacterium* spp.	1
*Comamonas* spp.	1
*Cupriavidus* spp.	5
*Delftia* spp.	2
*Herbaspirillum seropedicae*	1
*Pandoraea* spp.	1
*Pigmentiphaga* spp.	1
*Pseudomonas aeruginosa*	1
*Ralstonia* spp.	8
*Staphylococcus epidermidis*	1
*Stenotrophomonas* spp.	1
Unknown	16
TOTAL	306

a Numbers in parentheses indicate Thai strains; all other strains were isolated in the Northern Territory, Australia.

b Species assignment based on [28].

## Results

### Phenotypic Diversity of B. Ubonensis Isolates on ASA

**Figure 1:**
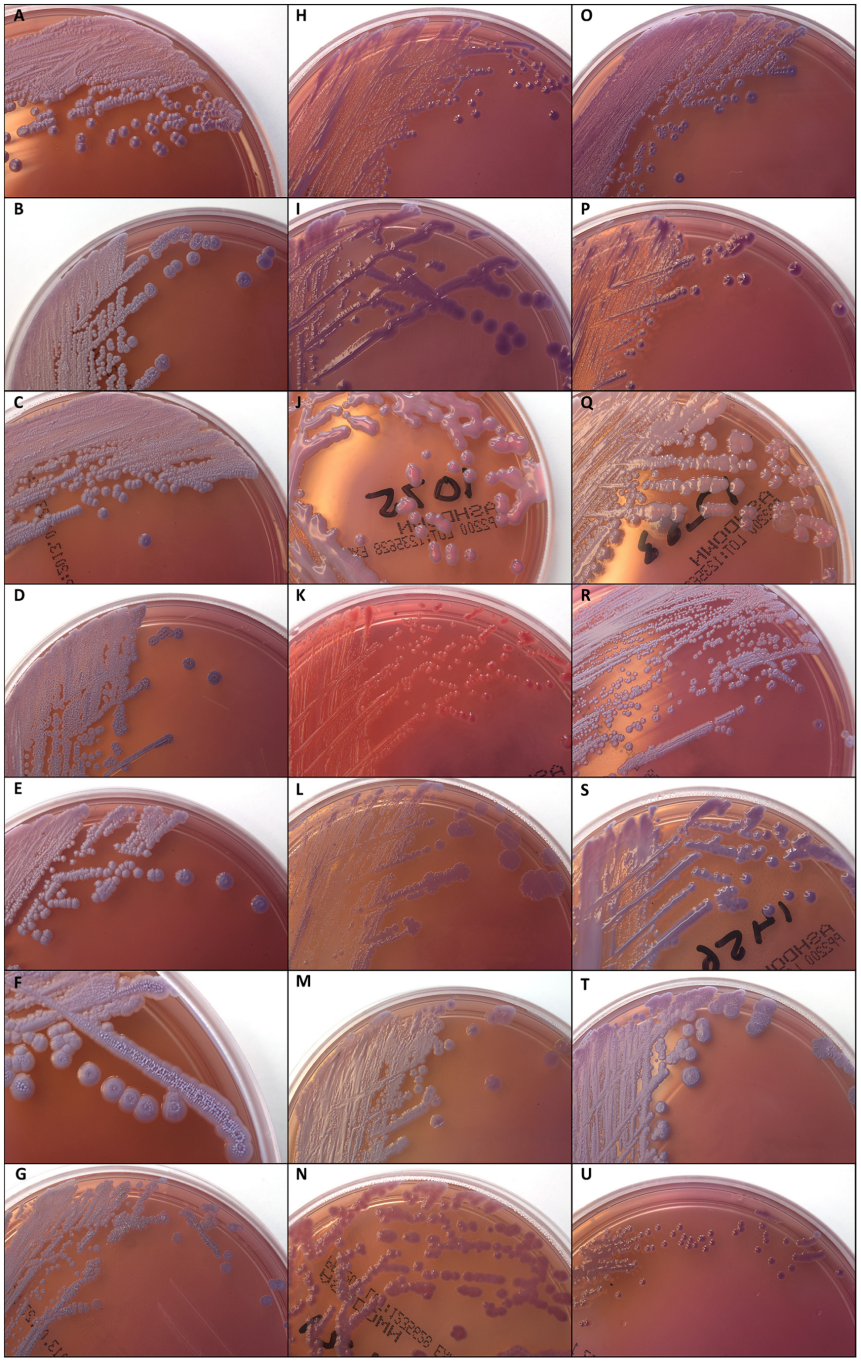
Colony morphologies of various *B. pseudomallei* near-neighbour species on Ashdown’s agar. Panels: A, *Burkholderia ubonensis* MSMB700; B, *B. ubonensis* MSMB704; C, *B. ubonensis* MSMB1138; D, *B. ubonensis* MSMB718; E, *B. ubonensis* MSMB1191; F, *B. ubonensis* MSMB1165; G, *B. ubonensis* MSMB1202; H, *Pandoraea* sp. MSMB824; I, *Herbaspirillum seropedicae* MSMB1000; J, *Burkholderia diffusa* MSMB1075; K, *Chryseobacterium* sp. MSMB1448; L, *Cupriavidus metallidurans* MSMB1495; M, *Burkholderia vietnamiensis* MSMB1224; N, *Burkholderia multivorans* MSMB1271; O, *Burkholderia pyrrocinia* MSMB1147; P, *Delftia* sp. MSMB943; Q, *Ralstonia mannitolilytica* MSMB1253; R, *Burkholderia thailandensis* MSMB1415; S, *Burkholderia cepacia* MSMB1456; T, *B. cepacia* MSMB1011; U, *Acidovorax caeni* MSMB1260. On this medium, *Burkholderia ubonensis* demonstrates similar morphological characteristics to its potentially deadly near-neighbour, *Burkholderia pseudomallei*, including uptake of crystal violet and neutral red, and wrinkling of colonies after ∼72 h growth [13], [24]. Molecular genotyping is therefore necessary for differentiation of *B. ubonensis* from other bacterial species that grow on Ashdown’s medium. Note the morphological differences among *B. ubonensis* strains; several morphotypes have also been observed in *B. pseudomallei* [24].

ASA is commonly used for isolation of *B. pseudomallei* from clinical and environmental isolates in endemic regions, and is commercially available in Australia. The isolates examined in our study contain a cross-section of isolates that grow on ASA but are TTS1-negative [19], and thus are not *B. pseudomallei*. Several species (e.g. *Burkholderia diffusa*, *Chryseobacterium* spp., *Delftia* spp., *Ralstonia* spp., *Cupriavidus* spp. and *Acidovorax* spp.) possessed morphologies (Figure 1) that were clearly distinguishable from the expected *B. pseudomallei* morphotypes [24]; in other cases, isolates were indistinguishable from *B. pseudomallei.* The latter category contained mostly *Burkholderia* spp., particularly *B. ubonensis*, which demonstrated multiple morphotypes, many of which resembled *B. pseudomallei* morphotypes (Figure 1). This inability to differentiate most *B. ubonensis* strains from *B. pseudomallei* on ASA provided the impetus for the rest of this study.

### Identification of a B. Ubonensis-specific Target from Whole-genome Sequence Data

Comparative whole-genome analysis of 19 *Burkholderia* spp., which included eight *B. ubonensis* strains, was performed using *B. ubonensis* MSMB170 as the reference genome. With this approach, we identified a candidate 7 kb locus specific for *B. ubonensis*, corresponding to a region within *B. ubonensis* Bu contig PMP6xxBUBxxBu-101 (GenBank: ABBE01000101.1). Nucleotide BLAST analysis (http://blast.ncbi.nlm.nih.gov/) of 9,404 complete or draft microbial genomes confirmed specificity of this locus, with only a single significant hit occurring in *B. ubonensis* Bu (analysis performed 26-Mar-13).

### PCR Assay Design and Screening of the B. Ubonensis-specific Assay

**Figure 2:**
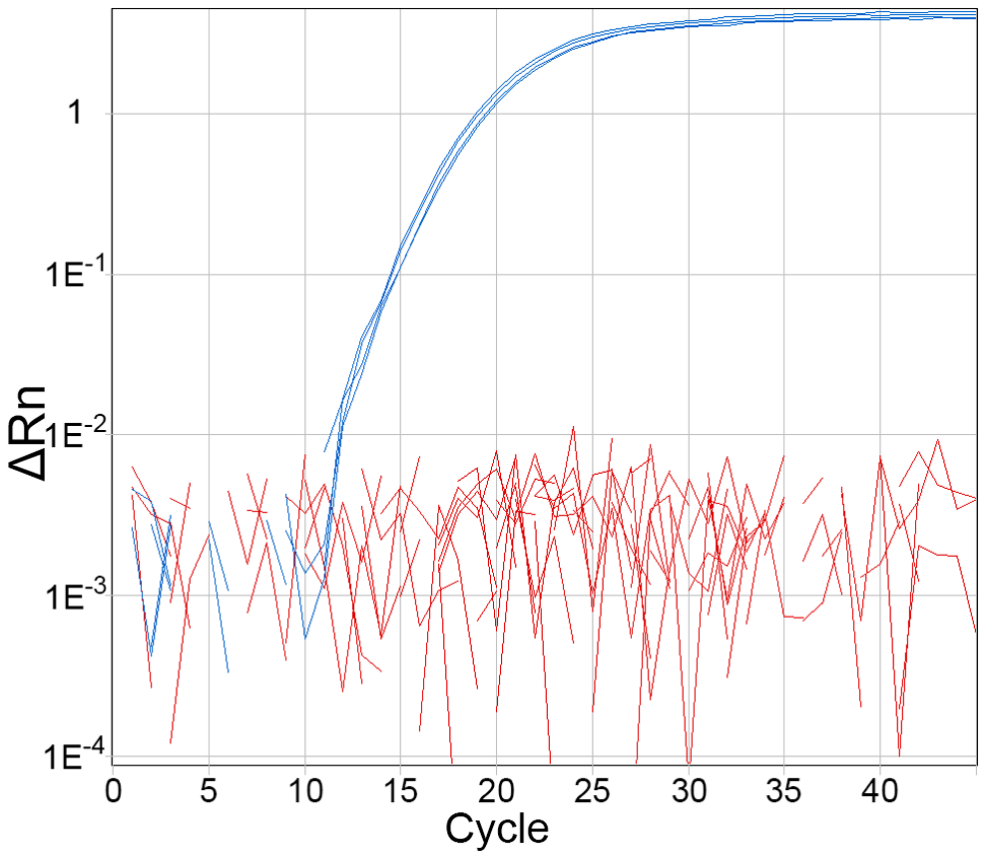
Bu550 *B. ubonensis-*specific real-time PCR. Bu550 differentiates *Burkholderia ubonensis* from other soil- and water-borne bacterial species that grow on Ashdown’s agar [13]. Only *B. ubonensis* (shown in blue) amplifies with this assay. Other species (shown in red) fail to amplify.

A Black Hole Quencher (BHQ) probe-based real-time PCR assay [25] was designed based on a conserved region within the 7 kb *B. ubonensis-*specific locus, encoding the hypothetical protein BuboB_03639. We chose BHQ probe technology due to its comparatively low cost compared with conventional TaqMan minor groove-binding probes (Applied Biosystems). In addition, the Bu550 assay can be multiplexed in a single PCR tube with the existing *B. pseudomallei* TTS1 BHQ assay [19] due to compatible CAL Fluor 540 and 6FAM fluorophore chemistries. The Bu550 assay was screened for specificity using a diverse panel of bacterial species that grow on ASA, with greatest representation of *B. ubonensis* and *B. pseudomallei* isolates (Table 1). In total, 306 isolates were tested, including 140 *B. ubonensis* and 86 *B. pseudomallei* isolates from Australia and Thailand. As predicted from *in silico* analyses, the Bu550 assay demonstrated 100% specificity for *B. ubonensis*, with all other examined species failing to amplify (Figure 2; Table 1). We obtained similar results with the TaqMan Environmental and Universal Master Mixes, although the Environmental Master Mix provided more robust amplification (data not shown).

## Discussion

Originally identified from soil collected in Ubon Ratchathani province, Thailand, in 1989 [16], *Burkholderia ubonensis* is now known to be an abundant environmental bacterium in northern Australia. Both regions are also endemic for *B. pseudomallei*, a pathogenic bacterium that causes significant morbidity and mortality, with up to 50% of infected individuals succumbing to disease [26]. Ashdown’s agar (ASA) is a common medium used in endemic regions to select *B. pseudomallei* from clinical and environmental specimens. However, ASA also supports the growth of other *Burkholderia* spp., several other Gram-negative bacteria and even some gentamicin-resistant strains of Gram-positive *Staphylococcus* spp. (Table 1; [27]). Of these non-*B. pseudomallei* species, we showed that *B. ubonensis* is the most frequently isolated in northern Australia due to its strong morphological resemblance to *B. pseudomallei*. Like *B. pseudomallei*, *B. ubonensis* possesses several morphotypes on ASA (Figure 1), many of which closely resemble *B. pseudomallei* morphotypes [24]. Therefore, it is impossible to differentiate *B. ubonensis* from *B. pseudomallei* based on morphological characteristics alone.

Due to the inherent difficulty in distinguishing *B. ubonensis* and *B. pseudomallei* based on morphology, we developed a cost-effective, rapid PCR-based method for differentiating *B. ubonensis* from other soil- and water-borne species that grow on ASA, particularly *B. pseudomallei*. Current methods of characterising *Burkholderia* spp. rely on dideoxy sequencing [8], [17], [18], which is an expensive and time-consuming endeavour for screening large isolate collections. We applied comparative whole-genome sequence analysis of 19 *Burkholderia* spp., including eight *B. ubonensis* isolates, to identify a locus specific for *B. ubonensis.* The specificity of one candidate locus, 550, was confirmed using in-depth *in silico* BLAST analysis. We subsequently developed a cost-effective real-time PCR assay, Bu550, for its interrogation. The performance of Bu550 was validated against a diverse collection of Burkholderiaceae and other bacterial species that grow on ASA, obtained from soil and water across the Northern Territory and Thailand. Our results indicate that Bu550 is highly specific towards *B. ubonensis*, with all other species we tested failing to amplify.

The design of the *B. ubonensis-*specific BHQ probe-based assay enables multiplex capability with the *B. pseudomallei*-specific assay, TTS1. *B. ubonensis* has demonstrated antagonistic activity towards *B. pseudomallei* and shows promise for biocontrol of naturally occurring *B. pseudomallei*, particularly in areas of high endemicity [15]. However, it is not yet known to what extent near-neighbour species such as *B. ubonensis* affect the natural prevalence of *B. pseudomallei*. Our future work will involve quantifying *B. ubonensis* and *B. pseudomallei* from soils obtained in *B. pseudomallei-*endemic regions to determine the relative abundance of these species, thereby improving our understanding of *B. pseudomallei* and *B. ubonensis* ecology. Bu550, in combination with TTS1, will be an important and valuable tool in such ecological studies.
